# Applying the updated MRC framework for developing and evaluating complex interventions with integrated implementation conceptual knowledge: an example using NeuroRehabilitation OnLine

**DOI:** 10.3389/frhs.2025.1562627

**Published:** 2025-05-06

**Authors:** Louise Connell, Suzanne Ackerley, Jo Rycroft-Malone

**Affiliations:** ^1^ Lancaster Medical School, Lancaster University, Lancaster, United Kingdom; ^2^ Rakehead Rehabilitation Centre, East Lancashire Hospitals NHS Trust, Burnley, Lancashire, United Kingdom

**Keywords:** context, sustainability, scale-up, determinant framework, ERIC strategies, Proctor's outcomes, implementation research logic model, rehabilitation

## Abstract

**Background:**

The updated 2021 UK Medical Research Council (MRC) Framework offers a valuable guide for implementation scientists to navigate the challenges of the development and evaluation of complex interventions. However, despite extensive citations, there is limited evidence of how the MRC Framework has been used in its entirety and limited integration with relevant implementation conceptual knowledge. To address this, we demonstrate the application of the updated MRC Framework incorporating implementation science frameworks, strategies, and outcomes. This example uses a telerehabilitation intervention, NeuroRehabilitation OnLine (NROL), implemented within an existing healthcare system.

**Methods:**

Within a clinical-academic partnership, we completed the MRC Framework checklist, and the context was described using the updated Consolidated Framework for Implementation Research (CFIR). We used a deliberative process to operationalise the MRC phases: adaptation of NROL based on the ADAPT guidance and establishing the feasibility of NROL through concurrent implementation and evaluation. Phases are described in two iterations: within a single service and then when scaled up as a regional innovation. Stakeholders were involved throughout. Implementation strategies were identified using the CFIR-Expert Recommendations for Implementing Change (CFIR-ERIC) matching tool. Proctor's implementation outcomes were selected for the evaluation.

**Results:**

The MRC Framework provided a useful structure when applied iteratively to address key uncertainties for implementation. Stakeholder co-production was integral to all phases, in both iterations. An additional sustainment phase was added to the framework, reflecting that the value proposition discussions with decision-makers inevitably culminated in decision points. This guided decision-making for NROL to be scaled up. Logic Models were co-produced and iterated to depict programme theory and formalise the integration of implementation conceptual knowledge.

**Conclusion:**

Synergistic in nature, the MRC Framework benefitted the conceptualisation of implementation through the use of its phases, and implementation science knowledge was useful in enacting the core elements within the MRC Framework. This example of application will be directly relevant to the field of rehabilitation and build transferable knowledge to enrich implementation research and practice.

## Background

The implementation-practice gap is well documented, with implementation scientists tasked with studying methods to promote the uptake of evidence-based practices. However, despite this important mission, the field faces significant criticisms. Most notably, these include its lack of relevance and timely application in implementation practice, with an overabundance of theories and frameworks, resulting in fragmented understanding, inconsistent use, and static rather than evolving theory, which all present barriers to advancing knowledge and practice (1–3).

**Figure 1:**
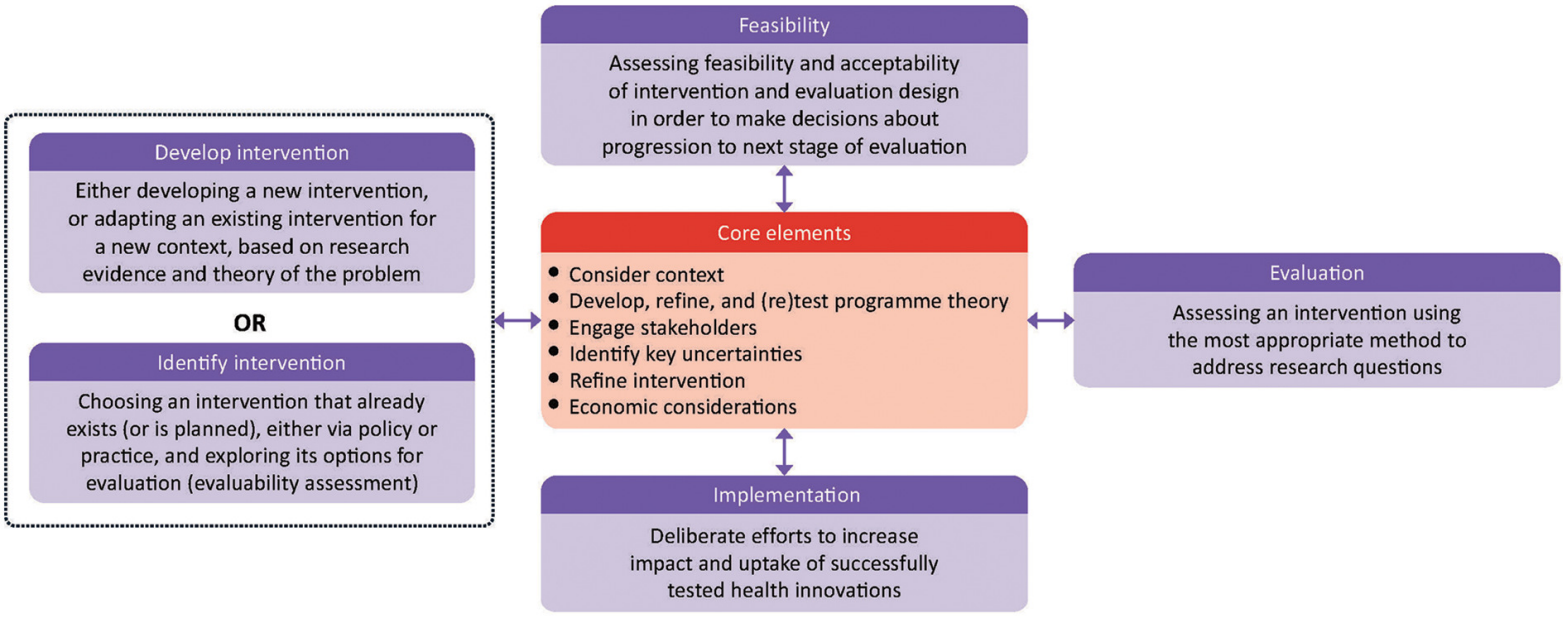
“Updated MRC Framework for developing and evaluating complex interventions”. Context, any feature of the circumstances in which an intervention is conceived, developed, evaluated, and implemented; programme theory, describes how an intervention is expected to lead to its effects and under what conditions—the programme theory should be tested and refined at all stages and used to guide the identification of uncertainties and research questions; stakeholders, those who are targeted by the intervention or policy, involved in its development or delivery, or more broadly those whose personal or professional interests are affected (that is, who have a stake in the topic)—this includes patients and members of the public as well as those linked in a professional capacity; uncertainties, identifying the key uncertainties that exist, given what is already known and what the programme theory, research team, and stakeholders identify as being most important to discover—these judgments inform the framing of research questions, which in turn govern the choice of research perspective; refinement, the process of fine tuning or making changes to the intervention once a preliminary version (prototype) has been developed; economic considerations, determining the comparative resource and outcome consequences of the interventions for those people and organisations affected. Reproduced with permission from “Framework for developing and evaluating complex interventions” by Skivington et al., licensed under CC BY 4.0.

In response to these challenges, this paper proposes the use of the updated United Kingdom (UK) Medical Research Council (MRC) Framework for developing and evaluating complex interventions (hereafter “MRC Framework”) as an overarching structure implementation scientists could use to guide their research (4–7). The MRC Framework was published in 2000 (4) with revision in 2006 (5) to offer a systematic architecture to the entire process of developing, evaluating, and implementing complex interventions. From 2006, the framework has consisted of non-linear phases, to develop/identify (adapt), assess feasibility, and implement and evaluate an intervention. The MRC Framework underwent substantial updates in 2021 (6), increasing its scope to a broader range of research perspectives (efficacy, effectiveness, theory-based, and systems) and to include six core elements that should be considered at each phase: identify key uncertainties, engage stakeholders, consider context, develop and refine programme theory, refine the intervention, and economic considerations (see Figure 1). The evolution of the framework reflects a growing sophistication and understanding of developing and evaluating complex interventions, providing a pluralistic guide for intervention implementation and evaluation. The MRC Framework is widely recognised and recommended, for example, with over 16,500 citations (8), and the UK National Institute for Health and Care Research recommends its use in their funding guidance (9). The guidance offers several case study examples (7), as does a recent discussion paper (10), but these are primarily of single phases. There is a need for further operational guidance with research that progresses through all phases of the framework (11).

The potential to combine the updated MRC Framework with developments in implementation science warrants consideration. There are many parallels between the MRC Framework and implementation research, including an emphasis on the importance of context and iteration and that implementation interventions tend to be complex interventions. There has been a call for the co-existence of multiple paradigms in the field of implementation science, with a plethora of theories, frameworks, and models that need to go beyond application with reverence (1). It is acknowledged that the MRC Framework, as a living document, should stimulate debate (12) and be used flexibly. Implementation conceptual knowledge has been combined previously (13–16), but to our knowledge, has not been integrated with the updated MRC Framework within an implementation research study. By leveraging the strengths of the MRC Framework and incorporating implementation science theories, a coherent, comprehensive approach to research could advance the field and better bridge the gap between implementation research and practice.

We provide a worked example of iteratively applying the updated MRC Framework to the implementation of a telerehabilitation intervention, NeuroRehabilitation OnLine (NROL), which, in the UK National Clinical Guideline for Stroke, is promoted as an exemplar innovation for delivering remote rehabilitation (17). NROL is a group-based multidisciplinary telerehabilitation approach for patients undergoing stroke and neurorehabilitation that has been implemented within the National Health System (NHS) in a region of the UK (18). Within rehabilitation literature, akin to wider health research, reference to the MRC Framework to date is predominantly related to methodology commentary or mentioned in passing without discussion on how the researchers applied the framework (19). We aim to fill a gap in evidence by using a real-world example of innovation implementation and scale-up of NROL to demonstrate the application of the updated MRC Framework with the integration of implementation science knowledge.

## Methods

The updated MRC Framework (Figure 1) was applied based on guidance (6, 7). We used a deliberative process to operationalise the MRC phases in their entirety: adaptation of NROL based on the ADAPT guidance (20) and establishing the feasibility of NROL through concurrent implementation and evaluation.

We completed the MRC Framework checklist (Table 1) (7) to expand on the core elements, and used implementation conceptual knowledge to help enact these, including the Consolidated Framework for Implementation Research (CFIR-CFIR) as a commonly used determinant framework for context that has been associated with effective implementation (21, 22), the CFIR-Expert Recommendations for Implementing Change (CFIR-ERIC) matching tool to match identified CFIR-based contextual factors to relevant key ERIC implementation strategies for further stakeholder engagement (23–26), and Proctor's outcomes as an established taxonomy to conceptualise implementation success and inform addressing key uncertainties (27). The inherent relationships were formalised, guided by the Implementation Research Logic Model (IRLM) (13, 14), to depict elements of programme theory [determinants (CFIR), strategies (ERIC), mechanisms, and outcomes (Proctor's)] and provide a clear, visual summary for diverse stakeholders. The production of a logic model aligns with programme theory as a core element of the MRC Framework. The process was iterative and refined based on ongoing stakeholder input.

**Table 1:** MRC Framework checklist for NROL.

Addressing uncertainties	
1. Aim(s)/purpose(s) of the intervention	The aim of NROL is to enhance opportunity and access to neurorehabilitation for people with stroke or other neurological conditions, optimising existing resources in a healthcare system.
2. Key uncertainties given existing evidence about the intervention and the context in which it will be tested or implemented?	A review of the literature indicated telerehabilitation, using evidence-based interventions, can provide comparable results to in-person therapy (45–47) and is recommended in UK clinical guidelines (17). NROL, a telerehabilitation approach developed by the National Hospital for Neurology and Neurosurgery and University College London, showed good preliminary results as a standalone pilot (28) and was identified as a candidate intervention.
	Key uncertainties centred around whether NROL could be embedded within an existing healthcare system.
3. Do the research questions and methods address the key uncertainties?	Questions (informed by implementation conceptual knowledge):
	• Which adaptations are required to fit the new context/s? • Which implementation strategies facilitate implementation? • Is NROL feasible (appropriate and acceptable) from patient, staff and service perspectives? • Can NROL be adopted, and is it acceptable from a regional systems perspective? • Should NROL be sustained, and what facilitates sustainment?
	The ADAPT guidance was used to adapt NROL for different contexts (20). Sequential implementation was undertaken using targeted strategies identified using the CFIR-ERIC matching tool (25, 26), and mixed-methods evaluations were used to understand selected Proctor’s implementation outcomes (appropriateness, acceptability, adoption, and sustainability) (27).
	Stakeholders remained involved throughout, including decision-makers who were key in progression decisions.
4. Does the choice of research perspective reflect the key uncertainties identified?	A systems-based perspective reflected the requirement to understand how NROL could fit with the services and systems.
Engaging stakeholders	
1. Have you engaged stakeholders in the design/identification of the intervention and the development of the research protocol?	This work was undertaken within an existing clinical-academic partnership, with the project team embedded in the NHS and stakeholders evolved over time. The clinical-academic partnership included clinical academics (Lancaster University), NHS management (Service leads), NHS therapy staff (deliverers, referrers), NROL Operational team (Manager, Technology Support, Administrator), healthcare decision-makers (including Commissioners), and academics. Other key stakeholder groups include patients and their families, third-sector organisations (SameYou Charity, Stroke Association), and the original standalone intervention developers (National Hospital for Neurology and Neurosurgery and University College London).
2. Have you engaged stakeholders in the conduct of the research and the dissemination of findings?	Stakeholders were identified and involved throughout. Decision-makers were involved from the outset and were key in decision points.
	The ERIC strategies and co-production activities used to jointly adapt, implement, and evaluate NROL are provided in Table 2, with further detail in the supplementary material.
3. Have all stakeholders declared any potential conflicts of interest?	None declared.
Considering context	
1. Have you identified all the dimensions of context that may influence how the intervention achieves its effects?	Context has an increasingly important focus within the updated MRC Framework. We used domains from the CFIR as a determinant framework, providing a comprehensive menu of constructs that have been associated with effective implementation (21, 22). These provided the platform for the selection of targeted implementation strategies.
2. Have you considered how context may affect the scaling up or scaling out of the intervention?	Throughout the process, the wider healthcare system context was considered, including systems-level working, digital, and workforce strategies. These were integral in how NROL was adapted, implemented, and expanded from a single service to a regional multi-service level. Outputs and resources were developed considering scaling out and up.
Developing and refining programme theory	
1. Have you developed a programme theory?	The programme theory was depicted visually guided by the Implementation Research Logic Model (13) for iterations at a single-service intervention and as a regional service innovation (Figure 3). Each was co-produced and revisited periodically. Models reflected the requirements of stakeholders and the resources available.
2. Have you updated the programme theory?	
Refining the intervention	
1. Have you refined the intervention so that it is optimised for the context in which it will be implemented?	Ongoing refinement was dynamic and guided by stakeholder engagement and co-production activities. Considerations from the ADAPT guidance (20) and evaluation data were used formatively throughout implementation.
2. Have you specified how far and in what ways the intervention can be refined during implementation?	“Core components” of NROL were agreed and articulated (18).
Economic considerations	
1. Have you considered whether or not the value of the evidence, in terms of informing future decision-making, justifies the cost?	The value of NROL was considered beyond cost, including the value to the patients, workforce, organisations (i.e., partnered NHS Trusts) and system (i.e., Regional Integrated Care System). The evaluation benefitted from an existing clinical-academic partnership and external funding.
2. Have you identified an economic evaluation framework that is appropriate to the expected outcomes of the intervention?	Decision-makers identified essential elements for reporting and for inclusion in business cases for long-term commissioning to secure sustainment. These included service delivery metrics and staffing efficiencies and productivity (e.g., travel savings in terms of both mileage and time, and capacity).

### Context

As a core element, context is described below, with considerations summarised in the MRC checklist (Table 1).

NROL is a group-based, real-time telerehabilitation approach with dedicated technology assistance. The approach was originally developed in 2020 as a standalone intervention by clinical academics in London in response to the pandemic (28). From 2021, the approach was integrated within the NHS in a region of the UK to complement in-person therapy. Targeted therapy and community peer-support groups are delivered within recurring 6-week blocks, with groups facilitated by multidisciplinary team members from the existing therapy workforce coordinated by an NROL operational team. For comprehensive detail please refer to the NROL description using the Template for Intervention Description and Replication (TIDieR) checklist (18).

NROL within the NHS was implemented in two iterations over 4 years, first as a single-service level and then expanded to a regional innovation. For the first iteration (January 2021–March 2022) (29), the single service was a community-based neurological rehabilitation service in the northwest of England (East Lancashire Hospitals NHS Trust) consisting of two teams, the NeuroRehabilitation Team and Stroke Therapy Team. For the second iteration (April 2022 to ongoing) (30), the regional neurological services included were community-based stroke and/or neurorehabilitation services from four NHS Trusts (i.e., partnered Trusts) within the Lancashire and South Cumbria Integrated Care System in the northwest of England (East Lancashire Hospitals NHS Trust, University Hospitals of Morecambe Bay NHS Foundation Trust, Blackpool Teaching Hospitals NHS Foundation Trust, and Lancashire and South Cumbria NHS Foundation Trust). All the services provide multidisciplinary rehabilitation across community settings to adults who have a sudden onset or progressive/intermittent neurological condition.

Stakeholder groups have evolved over time and have included a clinical-academic partnership including clinical academics (Lancaster University), NHS management (Service leads), NHS therapy staff (deliverers, referrers), NROL Operational team (Manager, Technology Support, administrator), healthcare decision-makers (including Commissioners), and academics. Other key stakeholder groups include patients and families, third-sector organisations (SameYou Charity and Stroke Association), and the original standalone intervention developers (National Hospital for Neurology and Neurosurgery and University College London).

### Stakeholder co-production

The application of the MRC Framework was guided by co-production principles, where power is shared, and all team members contribute their perspectives and skills (31, 32). We respected and valued everyone's knowledge equally, fostering reciprocal relationships where everyone benefits from collaboration. By prioritising relationship-building and maintaining open communication, we ensured this project remained grounded in real-world experiences and served the needs of all stakeholders relevant to the implementation of NROL. Adaptation, implementation, and evaluation involved stakeholder co-production activities, using targeted strategies identified using the CFIR-ERIC matching tool, with relevant CFIR domains and constructs discussed and agreed upon within the clinical-academic partnership.

### Positionality

Our clinical-academic project team, embedded within the UK NHS in the northwest of England, embodies a unique perspective shaped by the inclusion of dual clinical-academic roles since 2019. Led by two physiotherapists (LC, SA) with stroke rehabilitation clinical practice and research experience, and expertise in implementation science and complex intervention research. We acknowledge the influence of our backgrounds on our viewpoints, and our dual roles provide the opportunity to be a conduit between the research and clinical practice communities. As part of the NHS ecosystem, we capitalised on internal knowledge, emphasising the importance of partnership in all project phases and decisions, and did not have a pre-determined project endpoint at the start. One co-author (JR-M), an author of the MRC Framework and an implementation framework, provided oversight and critique from a methodological and theoretical perspective.

## Results

The MRC Framework was applied in two iterations, first as a single-service intervention (January 2021–March 2022) and then as a regional multi-service innovation (April 2022 to ongoing). An additional “Sustainment” phase was included, with key decision points identified.

**Figure 2:**
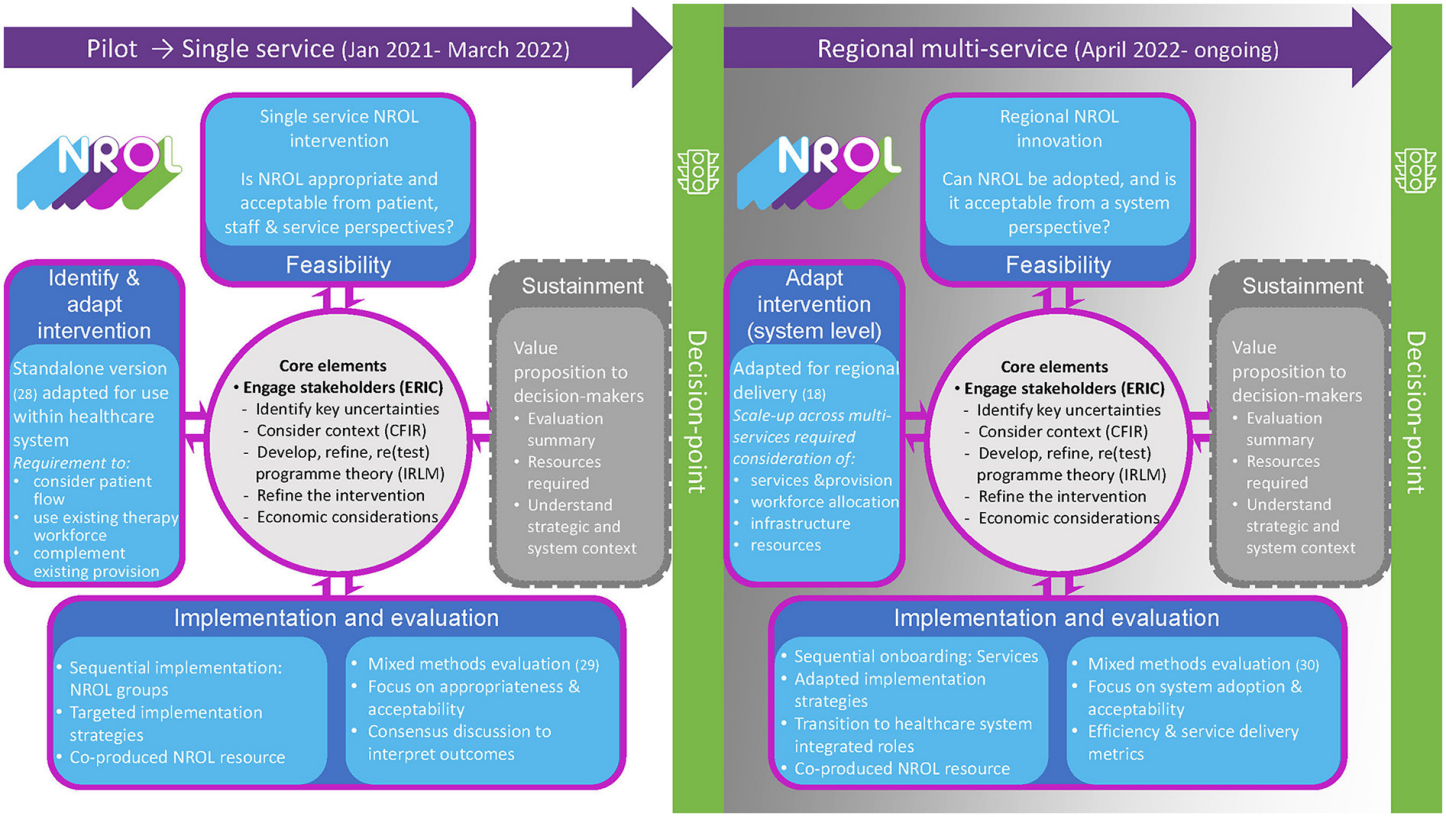
Advanced MRC Framework. Overview of the application of an advanced MRC Framework, showing two iterations: as a single-service intervention and regional multi-service innovation for NROL, NeuroRehabilitation OnLine. Advancements to the updated MRC include a sustainment phase. Integrated implementation conceptual knowledge includes ERIC, CFIR, and IRLM.

We have called our approach the “advanced” MRC Framework for ease of reference. An overview is shown in Figure 2 and detailed further below within the phase-specific considerations section.

### Stakeholder co-production

Key stakeholder groups engaged in multiple co-production activities to adapt, implement, and evaluate the intervention. These included identifying influential contextual factors, agreeing upon implementation strategies, selecting relevant implementation outcomes, and developing the logic models.

The contextual factors informed by the CFIR that were identified by stakeholders as influential during co-production activities were as follows:

- Characteristics of NROL: complexity, relative advantage, and adaptability.
- Outer setting: strategic fit, guidelines, pandemic, financial climate, and charitable funding.
- Inner setting (service and regional level): culture, leadership engagement, and available resources.
- Characteristics of individuals (patient and staff): knowledge and beliefs, self-efficacy, and capability.

**Figure 3:**
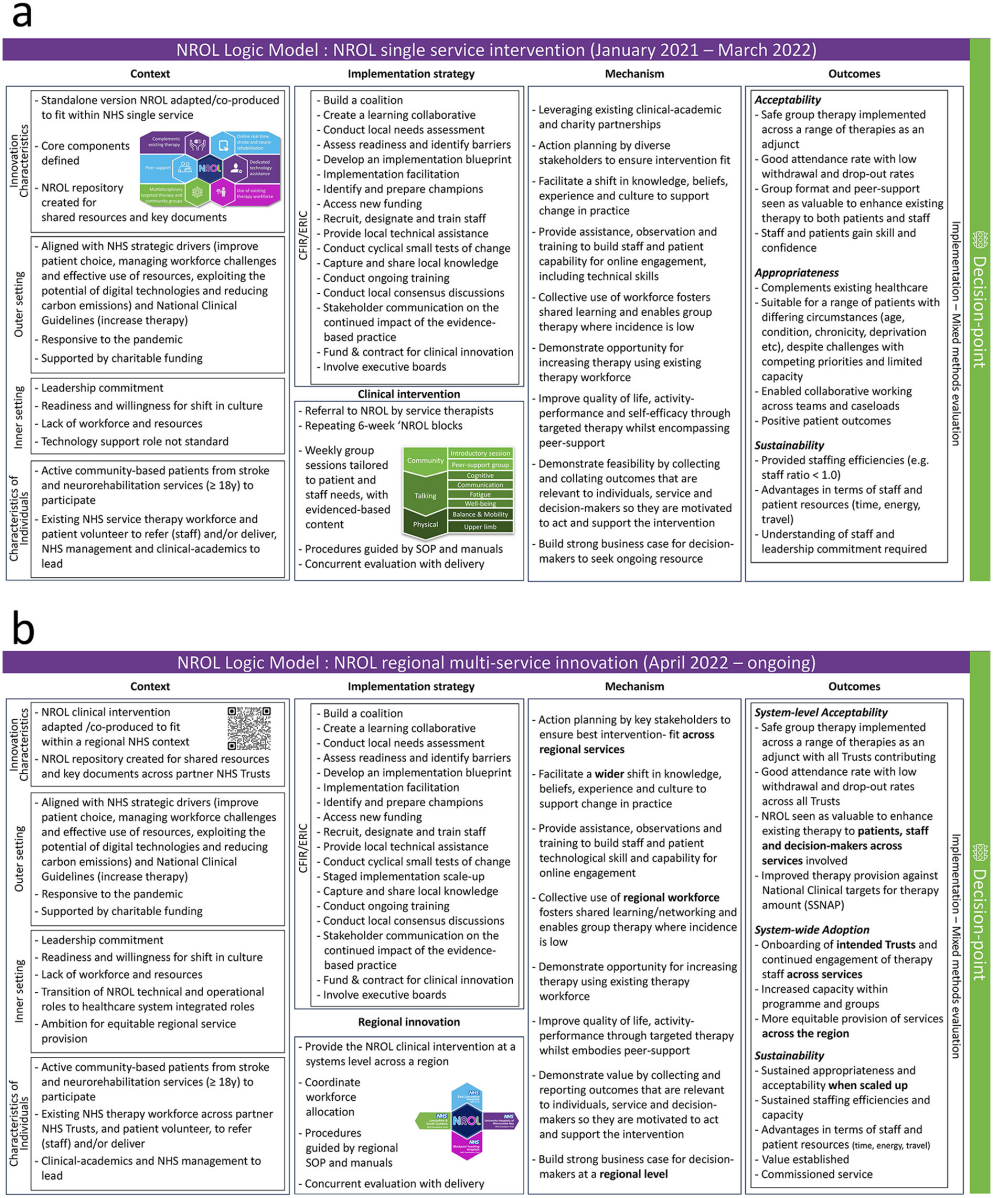
NROL Logic Models. Co-produced logic models showing two iterations: as a single-service intervention (**a**) and regional multi-service innovation (**b**). NROL, NeuroRehabilitation OnLine; NHS, National Health Service; CFIR, Consolidated Framework for Implementation Research; ERIC, Expert Recommendations for Implementing Change; SOP, standard operating procedure; SSNAP, Sentinel Stroke National Audit Programme.

Further details are provided within the NROL logic models (Figure 3) and in NROL publications (18, 29, 30).

The CFIR-ERIC matching tool identified 17 and 18 key ERIC strategies for the single-service and regional NROL iterations, respectively. Each co-production activity employed several of the ERIC strategies identified as important to support effective implementation. Illustrative examples of activities and contributions for each stakeholder group are provided in Table 2. Of note, the clinical-academic partnership was a key enabler of the co-production activities, as it was the basis of an established trusted relationship, facilitating meaningful stakeholder engagement with open and regular communication and supporting collaborative resource production and knowledge exchange.

**Table 2:** Stakeholder groups, co-production activities, and contributions.

Stakeholder group		Role	Examples of co-production activities	Examples of contribution
Clinical-academic partnership	Clinical academics	Instigated and co-led the adaptation, implementation, and evaluation	Co-led or involved in most co-production activities listed below	Integration of implementation science conceptual knowledge and evaluation mixed methods throughout.
				Robust evaluation to create a strong business case to support funding and sustainment
	NHS management (service leads)	Co-led integration into routine care	NROL management meetings (weekly initially, then monthly)	Strategic oversight led to the discussion and support for the expansion of NROL to regional innovation
			NROL champions meetings (quarterly with extra meetings around decision points)	
			Celebration events (*n* = 3)	
			Surveys (*n* = 5)	
			Co-production of standard operating procedures/NROL staff manual/TIDieR description (18)/logic models (Figure 3)	
			Co-authorship of NROL publications (30)	
	NHS therapy staff (deliverers, referrers)	Facilitate delivery of NROL, champions	Working group meetings (weekly)	Determined group capacity, which was iteratively progressed over time as they gained confidence in a new way of working
			NROL champions meetings (quarterly with extra meetings around decision points)	
			Celebration events (*n* = 3)	Therapy staff survey feedback prompted the revision of the referral process
			Surveys (*n* = 67) Interviews (*n* = 17)	
			Co-production of standard operating procedures/NROL staff manual/TIDieR description (18)/logic models (Figure 3)	
			Co-authorship of NROL publications (18, 29, 30)	
	NROL operational team (Manager, Technology Support, Administrator)	Plan and coordinate NROL delivery	NROL management meetings (weekly initially, then monthly)	Sustained and grown the buy-in from therapy staff across more organisations, with >75 staff delivering NROL and >200 staff observing sessions
			Working group meetings (weekly)	
			NROL champions meetings (quarterly with extra meetings around decision points)	
			Celebration events (*n* = 3)	
			Surveys (*n* = 3) Interviews (*n* = 3)	
			NROL video vignettes (*n* = 6) (48)	
			Co-production of standard operating procedures/NROL staff manual/TIDieR description (18)/logic models (Figure 3)	
			Co-authorship of NROL publications (30)	
	Healthcare decision-makers (Commissioners, system leaders)	Strategic input	Policy forums, and strategic discussions as required	Visibility at the executive level and secured funding opportunities
			Celebration events (*n* = 3)	
Other key stakeholders	Patients and their families	End-users, co-design of rehabilitation model, patient and public involvement, patient volunteers	NROL feedback sessions (after each block delivery)	Example modifications to NROL delivered to date include the addition of “Fatigue” and “Cognitive Strategy” groups, and the refinement of NROL resources (accessible)
			Co-production workshops (*n* = 6, 6–10 patients each)	
			Interviews (*n* = 13)	
			Focus groups (*n* = 2, 9 people, 1 h each)	
			Surveys (*n* = 176)	
			Regional patient and carer assurance groups (*n* = 2)	
			Facilitate delivery of NROL groups (*n* = 53)	
			NROL video vignettes (*n* = 6) (48)	
			Co-production of TIDieR description (18)/logic models (Figure 3)	
	Third-sector organisations (SameYou Charity, Stroke Association)	SameYou: Funder and co-creator of NROL pilot	Update meetings (monthly)	Initial investment and secured further funding that were fundamental to NROL implementation and sustainment
			Shared dissemination events (SameYou anniversary events, *n* = 3) and resources (49)	
			Activities to support future scale-up and sustainment (e.g., presentation to government and business consultancy meetings)	
		Stroke Association: Supporter and policy advocate	Organise Patient and Carer Assurance Group	Visibility at a policy level
			Support NROL session delivery for transition to Life after Stroke	
			Shared dissemination meetings (e.g., regional stroke meetings)	
	Standalone NROL intervention developers (National Hospital for Neurology and Neurosurgery and University College London)	Pilot NROL	Co-production meetings to adapt NROL (*n* = 4)	Input highlighted the importance of having technology support which facilitated role creation
			Sharing pilot evaluation reports (28)	
			Sharing of resources e.g., timetable system, staffing allocation	

### Phase-specific considerations

Operationalising the MRC Framework phases (see Figure 2) was dynamic and overlapping, and core elements were enacted using implementation concepts. A summary of the main considerations for the phases is provided below, highlighting the similarities and differences in focus between implementation at the single-service and regional levels and how these influenced working through the phases. For more detail, the phases have been described in a stepwise format together with relevant ERIC implementation strategies and involved stakeholder groups in the Supplementary Material.

#### Adaptation

The focus of this phase in both iterations was to ensure an intervention-context fit and plan for a successful implementation within the existing NHS healthcare system. The implementation strategies for both the single-service and regional levels had several similarities. Both prioritised building coalitions by leveraging existing clinical-academic partnerships and expanding involvement to include multiple stakeholders. Conducting local needs assessments and assessing readiness was integral, providing insights into alignment with strategic priorities, understanding context and contextual differences, and identifying staff and approval requirements. Initially, a learning collaborative was established, which agreed on implementation outcomes and co-produced a logic model for implementation in the single service (see Figure 3a). For the regional scale-up, the collaborative was extended to incorporate representatives from all the involved community-based neurological services from the four partnered NHS Trusts and management with overarching regional responsibility, and a new iteration of the logic model was co-produced for the regional multi-service approach (see Figure 3b).

#### Feasibility, implementation, and evaluation

To address the key uncertainties, NROL was sequentially implemented with targeted ERIC strategies. The similarities between the iterations included the acquisition of approvals, allocation of key roles, staff training, and undertaking of an evaluation with knowledge shared. In addition, accessing funding was crucial for initiating, sustaining, and scaling up operations. In the single-service iteration, efforts were concentrated locally, whereas, in the regional iteration, system-level coordination of services was required. This was needed to ensure overarching approvals and buy-in, formalisation of roles and responsibilities across services, and collective adoption. Evaluation in both iterations was mixed methods. A summary of the evaluation of NROL implemented at a single service has been published, with NROL demonstrating positive outcomes and opinions (29). The formative evaluation of the regional innovation had a shift in focus of implementation outcomes as agreed by the stakeholders with an emphasis of evaluation on understanding system-level efficiencies alongside delivery metrics (30).

#### Sustainability, sustainment, and decision point(s)

Sustainability can be defined as the “continued capacity to deliver the innovation, continued delivery of the innovation, and continued receipt of benefits” (33)*.* Sufficient emphasis should be placed on the factors that may contribute to sustainment throughout the process of adaptation, implementation, and evaluation, including the identification of facilitators, barriers and value. To reflect its importance, we deemed sustainment to warrant a distinct phase and added this in our use of the MRC Framework. The sustainment phase refers to when the intervention is supported and maintained with continued delivery, allowing for modifications and tailoring as necessary (34). Sustainability prioritised communication and relationships between stakeholders, including decision-makers, to facilitate the continued use and impact of the intervention. The coalition, in particular clinical-academic staff, management, and champions, played a pivotal role in strategic alignment and identifying opportunities for visibility. Funding and contracting in both iterations used evaluation findings to identify advantages, resources, and barriers, with the knowledge shared and interpreted by stakeholders. A key difference lay in the scale of stakeholder engagement. The regional approach extended the stakeholder group to include regional healthcare system commissioners and executive boards and required business cases.

It was recognised that framework phases are often time-limited by factors such as staffing and financial resources and inevitably culminate in decision points. For example, decisions had to be reached as to whether to move forward, and, e.g., how to continue current provision, discontinue, or scale up. Although decision points were evident in both iterations, differences in decision-makers reflected the widening scope, with engagement changing from service level to regional executive boards. In this example, a first major decision point was required at the end of single-service funding for NROL, and then again at a regional level (see Figure 2). The different strategic contexts altered the value proposition, with the initial decision point discussing the potential for NROL scale-up to a regional entity and the second reflecting the aspiration of commissioning for sustainment of the regional NROL innovation.

### Implementation research logic models

Elements of the programme theory were described visually using co-produced logic models, with final versions detailed for the single-service and regional multi-service iterations (Figure 3). These bring together the use of the CFIR and ERIC strategies, and implementation outcomes and highlight the shift in focus to the system level when NROL was scaled up to a regional level.

## Discussion

This publication presents a novel approach to the use of the MRC Framework in combination with implementation conceptual knowledge. To advance the MRC Framework, we integrated the CFIR, ERIC strategies, and selected Proctor's implementation outcomes as methods to enact several of the framework's core elements. This was collated and visualised guided by Implementation Research Logic Models. In addition, we incorporated a focus on sustainability by adding a distinct phase dedicated to sustainment. Furthermore, we acknowledge the existence of decision points that may lead to new iterations of the MRC Framework application, facilitating further progression through the phases to scale up an intervention. This example should assist implementation research and practice by demonstrating how paradigms can be combined, providing a concrete illustration of the application of *all* phases of the MRC Framework as an intervention is scaled up and offering a valuable reference for future work.

A challenge of the MRC Framework is that it lacks some operational guidance, with core elements that need to be considered at each phase stated but with minimal detail as to how to apply these. We have provided an example of how the operationalisation of the framework could be achieved. The MRC Framework provided an overarching document for framing stakeholder co-production activities around essential considerations for adaptation, implementation, and evaluation. As encouraged (7, 10), we completed an MRC checklist and updated this over time, with the optimal timing and level of detail of this checklist guided by pragmatic decisions and resources. In agreement with the recommendation by Levack et al., we found the checklist a useful tool to frame our research methods. Integrating implementation conceptual knowledge was helpful, particularly regarding the core elements of “considering context,” “engaging stakeholders,” and “developing and refining programme theory.” The iterative nature of the MRC Framework allowed for responsiveness to emerging challenges and opportunities, but we also highlighted the need for clear decision points regarding intervention continuation, modification, or cessation based on the value proposition, including evaluation outcomes.

Responding to suggestions by others (7, 10, 19), we aimed to incorporate a tangible, usable example illustrating the application of the framework. However, it is unclear whether the process or reporting enhanced the effectiveness of our implementation efforts and offset the resources, time, and expertise required, or simply increased the complexity involved. The process of grappling with this example prompted us to consider implementation and sustainability factors early in our planning process but also facilitated a more systematic process that likely improved our overall approach and helped in building stakeholder relationships. This tension between the use of theory-driven methods vs. practical usefulness is a challenge in many areas of implementation research (2). However, we feel the trade-off in resources may yield further benefits in future iterations, scale-out, and subsequent applications in other settings and topics.

Acknowledging the complementary nature, and distinctions, between implementation and sustainability is imperative in ensuring the longevity of interventions within complex systems. Sustainability has gained increasing consideration in implementation science. The updated MRC Framework (7) places limited direct emphasis on sustainability, with the exception that the ADAPT guidance (20) is highlighted in the adaptation phase, and implementing and maintaining the intervention at scale is mentioned. Implementation and sustainment are interconnected and need to be planned for; therefore, there is potential for the MRC Framework to further embrace sustainment perspectives. It is noted that sustainment typically places a heavier emphasis on factors within the outer setting, navigating beyond the immediate organisational boundaries and encompassing socioeconomic, cultural, environmental, and strategic contexts (35, 36). These factors encompass elements such as policy alignment, community engagement, resource availability, and the long-term commitment of stakeholders, extending the scope of considerations beyond the implementation phase. Recognising the increased attention on sustainability, alongside existing implementation frameworks, presents an opportunity to build on established knowledge rather than “reinvent the wheel” (1, 2). We deemed the integration of sustainment into the MRC Framework was sufficient and avoided introducing another framework and its language.

Our systems-based perspective reflected the requirement to understand how the intervention/innovation could fit with a system/s, with principles of co-production through a clinical-academic partnership paramount. What contributes to co-production is difficult to define. We have given some examples of co-production activities but acknowledge that not all activities are tangible. Co-production and implementation are more processes than a list of activities, hindering replication. Work by Metz et al. and others has highlighted the importance of relational aspects in implementation (37–39). Arguably the “who” might be equally as important as the “what.” In our advanced MRC framework (Figure 2), we have reflected the importance of stakeholder engagement as an overarching principle when considering the remaining core elements.

This example demonstrates progression through adaptation, implementation, evaluation, and sustainment phases, and the clinical-academic partnership was a key mechanism in our successful evolution. Specifically, it was significant that the clinical-academic project team was embedded in the NHS, and they were able to leverage internal knowledge and take opportunistic approaches to inform key decision-makers. When the decision to scale up the intervention to a regional level was made, stakeholders were broadened and arguably influenced by pre-existing relationships and credibility. The time and effort for this relational engagement is significant, though it is often not described or resourced with the advantages taken for granted, which has implications for generalisability. With research co-production a neglected pathway to impact (39), there is a key role for clinical-academic partnerships in driving the implementation and evaluation of complex interventions which warrants further exploration and recognition.

We needed to involve the right people with insight and authority, supplied with the right knowledge, to enable decisions on sustainment. The dynamic nature of real-world implementation meant that a sustainability outcome was not always predictable, and this uncertainty necessitated continual flexibility in our approach. The concept of value proposition was included as a new addition to this advanced MRC Framework within the sustainment phase. This was provided by the components of an evaluation summary and by the resource and strategy overview tailored to this phase to enable an informed discussion. Further, deciding when and how to move on to the next phases within the MRC Framework, especially given phase overlap, can be hard to determine. The MRC Framework acknowledges that there are often trade-offs between precise, narrow-focused answers and broader, complex inquiries and “that at any phase key core elements should be considered to guide decisions as to whether or not the research should proceed to the next phase, return to a previous phase, repeat a phase or be aborted” (7)*.* This is described most clearly in the feasibility phase, with progression criteria now generally a given within feasibility protocols. Our example demonstrated an addition with clear major decision points, a crucial juncture where decisions needed to be made about whether to continue, modify, expand, or stop the intervention based on evaluations and available information.

### Limitations

While our utilisation of the MRC Framework provided a structured architecture to guide our implementation efforts, it is important to acknowledge the limitations and challenges encountered during its application. We applied the MRC core elements to varying degrees; for example, we had a limited focus on health economics. Minimal resources were available for this, and ongoing work has secured funding to undertake further economic analysis. There is a dependency here, in that the resource for the health economic analysis was not available until the acceptability and value of the intervention had been demonstrated, but this is being increasingly focused on by decision-makers and may become crucial for decision points going forward. Our use of the MRC Framework involved modification. We merged, tweaked, and sometimes even diverged from the reported phases to suit the needs of our context and stakeholders, as implementation evidence would guide us to do. This flexibility, while necessary for practical application, introduced variability in adherence to the framework, though arguably it was used flexibly as intended. We included implementation conceptual knowledge within our advanced MRC Framework, such as the CFIR, ERIC, and Proctor's outcomes, but other implementation knowledge could equally be used, for example, a framework to describe context, such as the Implementation in Context (ICON) Framework (40). Similarly, other evaluation frameworks, such as RE-AIM or PRECEDE-PROCEED, or broader frameworks, such as the Exploration, Preparation, Implementation, Sustainment (EPIS) Framework, could be used (16, 41–44). We invite others to try applying the MRC Framework in their implementation efforts and provide feedback on their experiences, and we will continue to refine our work for other regions and clinical areas as it progresses.

## Conclusion

In conclusion, our study contributes to the implementation science literature by demonstrating a novel integration of implementation knowledge with the MRC Framework, providing an example of the original and revelatory use of these related but separate evidence bases and answering the call to advance and evolve existing frameworks rather than duplicating existing evidence (1, 2, 7, 22). By addressing key challenges collectively within a clinical-academic partnership, we provide insights that can inform future research and practice in the field of implementation science and practice. Our experience underscores the importance of balancing theoretical frameworks with practical considerations to ensure relevance and effectiveness in implementation science endeavours. This application example will be directly relevant to the field of rehabilitation and build transferable knowledge to enrich implementation research and practice.

## Data Availability

The raw data supporting the conclusions of this article will be made available by the authors, without undue reservation.
