# Supplementary material for: Applying the updated MRC framework for developing and evaluating complex interventions with integrated implementation conceptual knowledge: an example using NeuroRehabilitation OnLine
Source: Front Health Serv. 2025 May 6;5:1562627. doi: 10.3389/frhs.2025.1562627 (PMC12089040; doi:10.3389/frhs.2025.1562627)
Supplement: Supplementary file 1 [file Table1.pdf]

Supplementary Table 1: MRC framework phase-specific implementation strategies, considerations and stakeholders

ERIC strategy	Single-service (January 2021 – March 2022)	Stakeholder group	ERIC strategy	Regional multi-service (April 2022 – ongoing)	Stakeholder group
Identification and Adaptation phase					
Build a coalition	Leveraging existing clinical-academic relationships and charity partnerships the concept of NROL within the NHS was cultivated.	Clinical-academics<br>NHS Management<br>Third sector organisations (SameYou, Stroke Association)<br>Standalone intervention developers	Build a coalition	Involvement was extended to include leaders at NHS Trusts across the region who provide community-based stroke and/or neurorehabilitation services (i.e. partnered NHS Trusts), and management with overarching responsibility within the regional integrated care system.	NROL Operational team<br>NHS Management<br>Therapy staff<br>Third sector organisations (SameYou, Stroke Association)
Conduct local needs assessment	Identified that NROL aligned with local and national strategic priorities (1-5) and provided scope to help meet clinical guidelines (6).<br>Understanding gained of pilot context compared to local single service NHS context.	Clinical-academics<br>NHS Management<br>Therapy staff<br>Academics	Conduct local needs assessment	Identified that NROL aligned with local and national strategic priorities (1-5) and provided scope to help meet clinical guidelines (6).<br>Understanding gained of partnered NHS Trusts service/s contexts (e.g. multi-service) given differences in service structure, remit and provision.	Therapy staff<br>Clinical-academics<br>NHS Management<br>Academics
Assess readiness and identify barriers	Consensus on contextual factors that are influential for implementation. Need for local information governance and data protection approvals.<br>Technology assistance deemed essential but role not standard within service.	Clinical-academics<br>NHS Management	Assess readiness and identify barriers	Consensus on contextual factors that are influential for implementation. Need for regional information governance and data protection approvals.	Clinical-academics<br>NHS Management
Create a learning collaborative	A learning collaborative was established and evolved over time as NROL was expanded. It involved diverse stakeholders such as patients, healthcare system staff (therapy	Clinical-academics<br>NHS Management	Create a learning collaborative	The learning collaborative was extended to ensure representation across the region.	Clinical-academics<br>NHS Management<br>NROL Operational team

	staff, management, administrative and decision-makers), clinical-academic and academic staff, third sector organisations and the original intervention developers).				
Develop an implementation blueprint	An Implementation Research Logic Model, and NROL Standard Operating Procedure (SOP) and materials were co-produced and revised periodically across phases.	Clinical-academics<br>NHS Management<br>Therapy staff<br>Patients & families	Develop an implementation blueprint	A new iteration of the Implementation Research Logic Model was co-produced for regional delivery. NROL SOP and materials revised for multi-service approach e.g. Change in referral process, staffing etc.	Clinical-academics<br>NHS Management<br>NROL Operational team<br>Therapy staff<br>Patients & families
Feasibility, Implementation and Evaluation phase					
Implementation facilitation	Interactive problem solving and discussion to support decision-making.<br>Facilitated local information governance and data protection approvals.	Clinical-academics<br>NHS Management<br>NROL Operational team<br>Therapy staff<br>Standalone intervention developers<br>Patients & families	Implementation facilitation	Interactive problem solving and discussion to support decision-making.<br>Sought support from regional stakeholders to obtain information regional governance and data protection approvals	Clinical-academics<br>NHS Management<br>NROL Operational team<br>Therapy staff<br>Patients & families
Access new funding	Charity funding for key NROL technical, operational and evaluation roles.	Clinical-academics<br>Third sector organisations (SameYou)	Access new funding	Charity and NHS England funding for expanding in regional services and on-going technical, operational and evaluation roles	Clinical-academics<br>Third sector organisations (SameYou)<br>NHS Management<br>NROL Operational team
Recruit, designate and train	Recruit/designate therapy staff to deliver groups, including new technical role.	Clinical-academics	Recruit, designate and train	Transition of NROL technical and operational roles to healthcare system integrated roles, train therapy staff from partnered NHS Trusts.	Clinical-academics<br>NHS Management<br>NROL Operational team

Identify and prepare champion	Single-service champions developed organically from learning collaborative and those delivering therapy.	Clinical-academics<br><br>NHS Management	Identify and prepare champion	Champions identified from each service in the region involved in community-based stroke and/or neurorehabilitation delivery.	NHS Management
Provide local technical assistance	Technical assistance for patients and staff.	Clinical-academics<br>NROL Operational team	Provide local technical assistance	Technical assistance for patients and staff	NROL Operational team
Conduct cyclical small tests of change	Feasibility of the adapted NROL was determined through an initial trial (single group, small cohort), and then sequential implementation (broader range of multidisciplinary therapy groups, increasing patient cohort) and evaluation at a single service. NROL was refined iteratively e.g. adding/trialling new groups.	Clinical-academics<br>Therapy staff<br>Patients & families	Conduct cyclical small tests of change	Feasibility of regional adoption was determined through sequential implementation of Trusts and concurrent evaluation at a multi-service. NROL was refined iteratively in response to stakeholder input.	Clinical-academics<br>Therapy staff<br>Patients & families
			Staged implementation scale-up	Sequential onboarding of regional community-based stroke and neurorehabilitation services.<br>Feasibility was determined through concurrent implementation and evaluation at a multi-service systems level.	Clinical-academics<br>NROL Operational team
Capture and share local knowledge	Collected and collated quantitative and qualitative patient, staff and service data.<br>Shared with learning collaborative, champions and wider stakeholders at regular meetings.	Clinical-academics<br>NHS Management<br>NROL Operational team<br>Patients & families<br>Standalone intervention developers<br>Third sector organisations (SameYou, Stroke Association)	Capture and share local knowledge	Collected and collated quantitative and qualitative patient, staff and service data. Focus on further understanding efficiencies.<br>Shared with learning collaborative, champions and wider stakeholders at regular meetings.	Clinical-academics<br>NHS Management<br>NROL Operational team<br>Patients & families<br>Third sector organisations (SameYou, Stroke Association)
Conduct ongoing training	Observations enabled therapists and other staff to gain understanding of	Clinical-academics	Conduct ongoing training	Observations enabled therapists and other staff to gain understanding of	Clinical-academics

	NROL, and experience groups. This could be for insight or experiential learning before facilitating groups.	NROL Operational team<br>Therapy staff		NROL, and experience groups. This could be for insight or experiential learning before facilitating groups.	NROL Operational team<br>Therapy staff
Conduct local consensus discussions	Implementation outcomes were discussed, and consensus reached on patient, staff and service acceptability, and appropriateness of NROL within the service.	Clinical-academics<br>NHS Management<br>Patients & families	Conduct local consensus discussions	At a regional level, consensus was reached regarding system adoption and acceptability.	Clinical-academics<br>NHS Management<br>NROL Operational team<br>Patients & families
Sustainment phase					
Stakeholder communication on the continued impact of the evidence-based practice	Continued engagement with local clinical, service and regional leads for championing NROL and communicating evaluation findings. The coalition and champions were key in strategic alignment and highlighting opportunities for visibility and impact.	Clinical-academics<br>NHS Management<br>Academics<br>Third sector organisations (SameYou)	Stakeholder communication on the continued impact of the evidence-based practice	Continued engagement with regional clinical, service and regional leads for championing NROL and communicating evaluation findings. The coalition and champions were key in strategic alignment and highlighting opportunities for visibility and impact.	Clinical-academics<br>NHS Management<br>NROL Operational team<br>Academics<br>Third sector organisations (SameYou)
Fund and contract for clinical innovation	Identified advantages in terms of staff and patient resources, barriers and facilitators identified.	Clinical-academics<br>NHS Management	Fund and contract for clinical innovation	Developed business case for commissioners with advantages identified in terms of staff and patient resources, barriers and facilitators identified.	Clinical-academics<br>NHS Management<br>Academics<br>Third sector organisations (SameYou)
Involve executive boards	Potential value proposition of local versus regional delivery discussed with decision-makers, outlining outcomes and resources required and tailoring to strategic priorities.	Clinical-academics<br>NHS Management<br>Healthcare decision-makers	Involve executive boards	Value proposition shared with decision-makers, outlining outcomes and resources required and tailoring to strategic priorities.	Clinical-academics<br>NHS Management<br>NROL Operational team<br>Healthcare decision-makers

References

1. NHS Lancashire and South Cumbria Integrated Care Board. Lancashire and South Cumbria Integrated Care System: Our NHS Joint Forward Plan from 2023 onwards. 2023 [Available from: https://www.healthierlsc.co.uk/application/files/7416/8977/5384/LSC_ICB_Joint_Forward_Plan_2023.pdf.
2. NHS England. 2022/23 priorities and operational planning guidance. 2022 [Available from: https://www.england.nhs.uk/wp-content/uploads/2022/02/20211223-B1160-2022-23-priorities-and-operational-planning-guidance-v3.2.pdf.

3. NHS England. 2023/24 priorities and operational planning guidance 2023 [Available from: https://www.england.nhs.uk/wp-content/uploads/2022/12/PRN00021-23-24-priorities-and-operational-planning-guidance-v1.1.pdf.
4. NHS England. The NHS Long Term Plan. 2019 [Available from: https://www.england.nhs.uk/wp-content/uploads/2022/07/nhs-long-term-plan-version-1.2.pdf.
5. NHS England. NHS Long Term Workforce Plan. 2023 [Available from: https://www.england.nhs.uk/wp-content/uploads/2023/06/nhs-long-term-workforce-plan-v1.21.pdf.
6. Royal College of Physicians. National Clinical Guideline for the Stroke for the United Kingdom and Ireland. 2023 [Available from: https://www.strokeguideline.org/app/uploads/2023/04/National-Clinical-Guideline-for-Stroke-2023.pdf.
